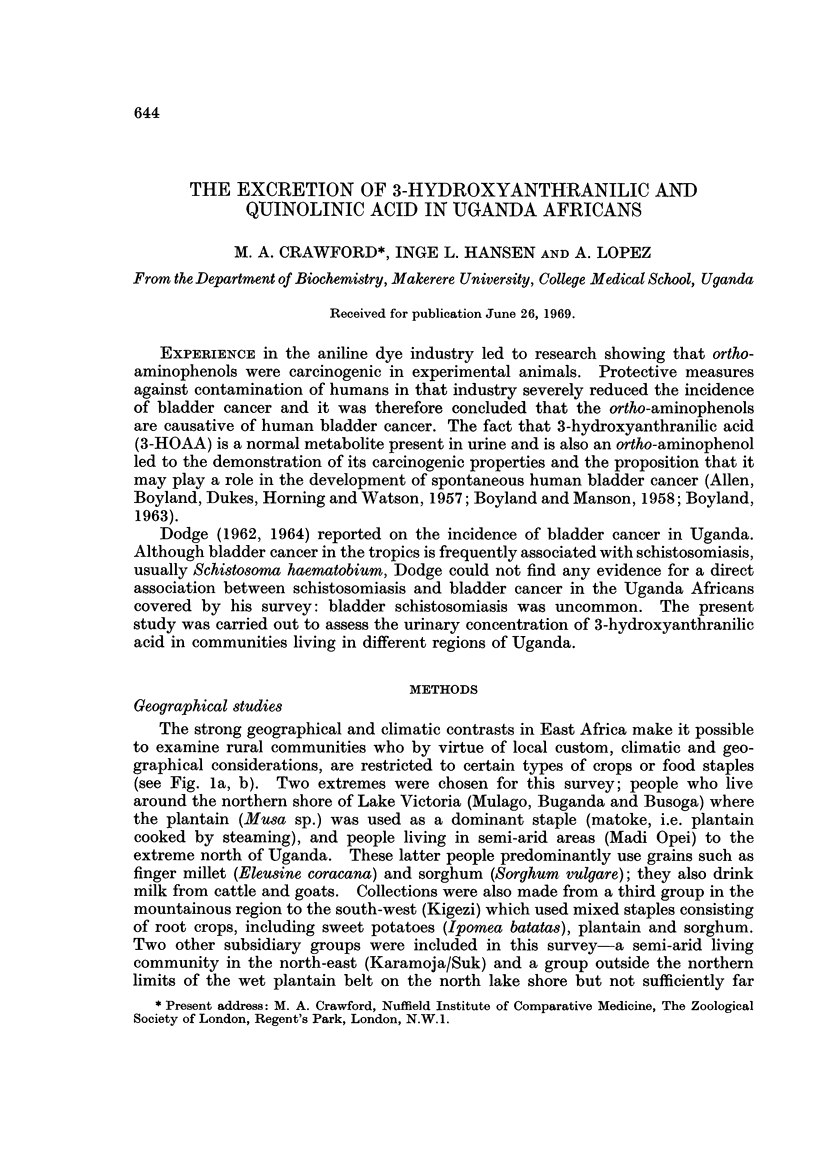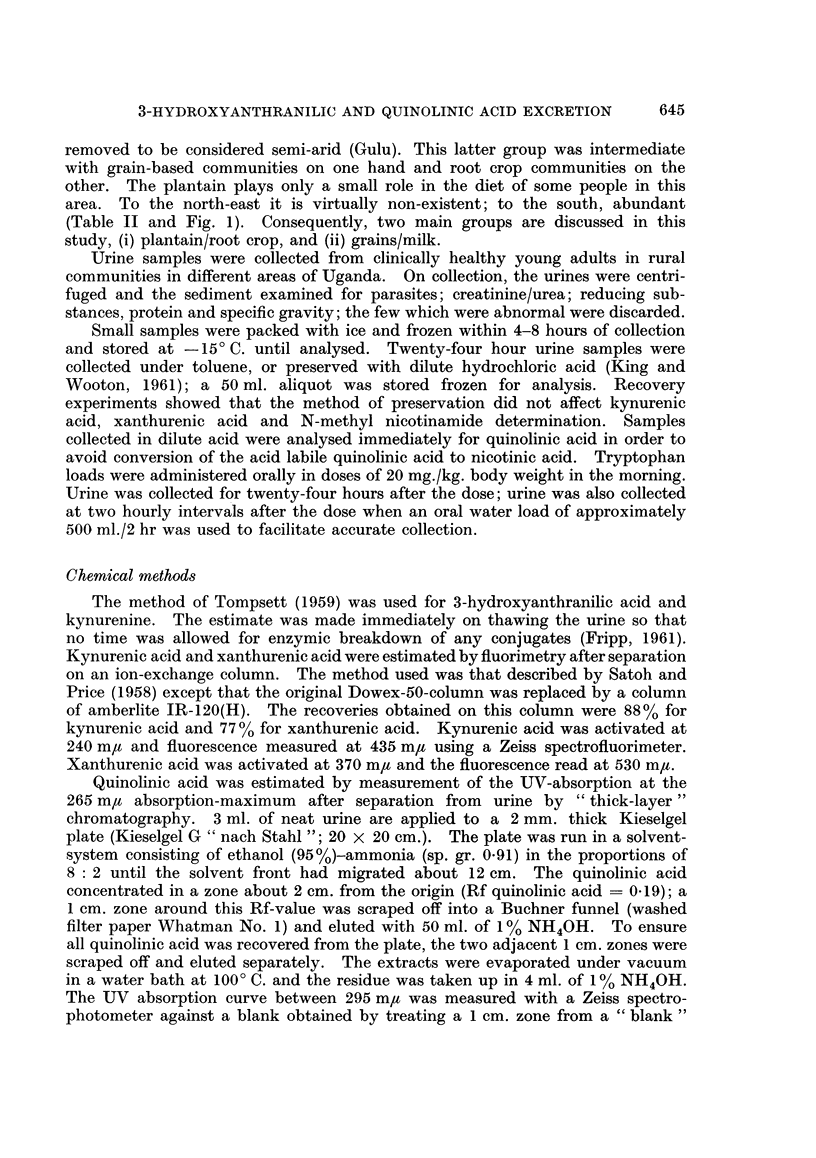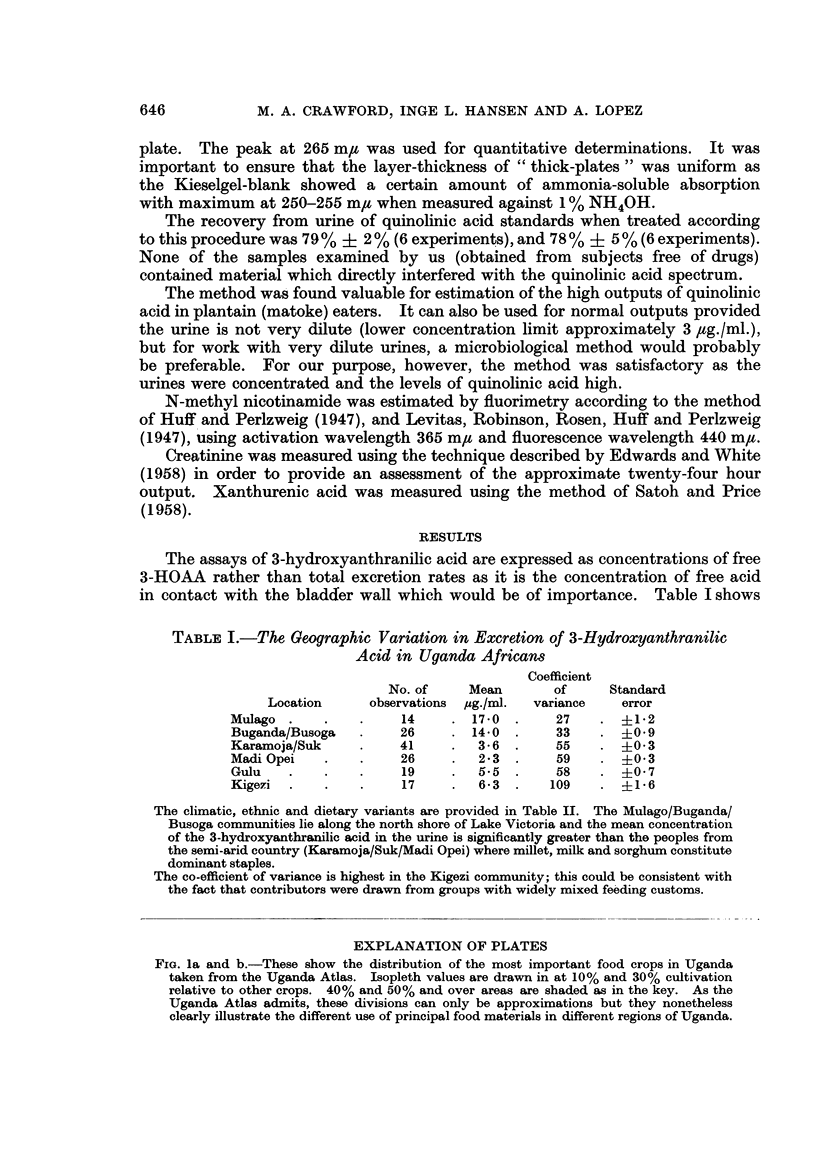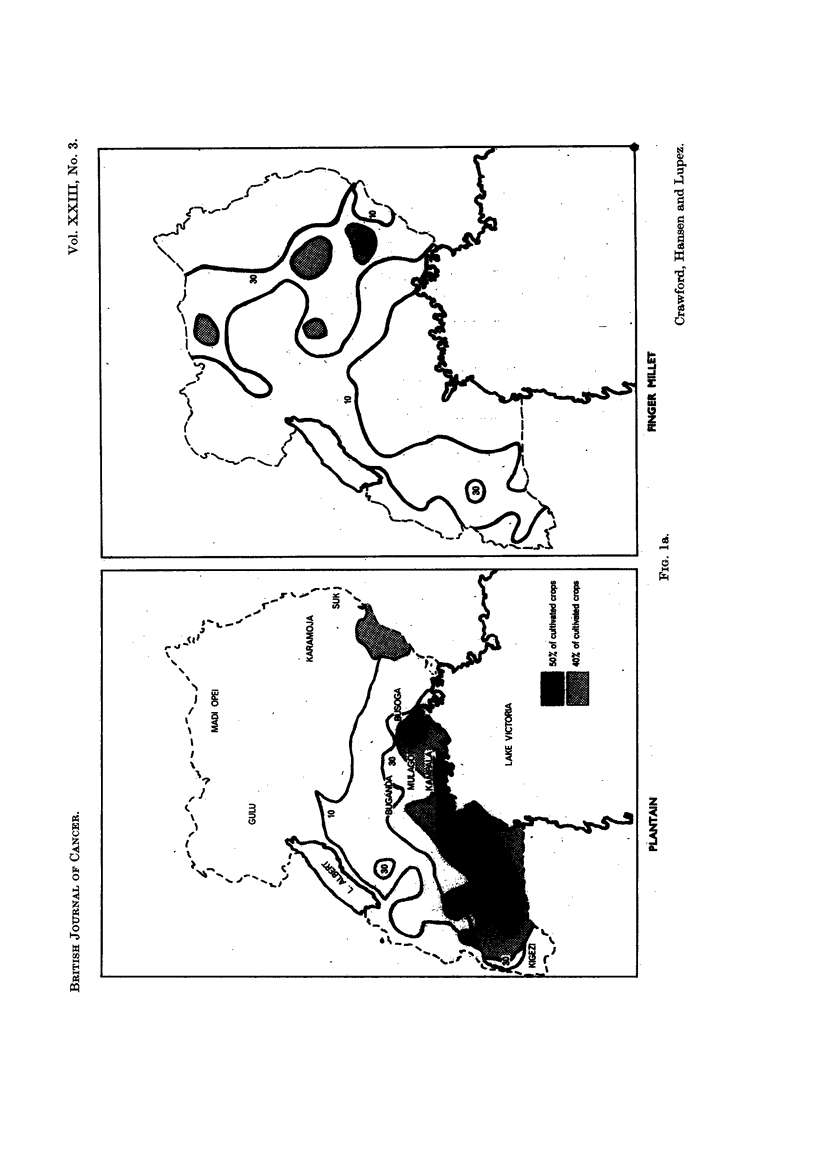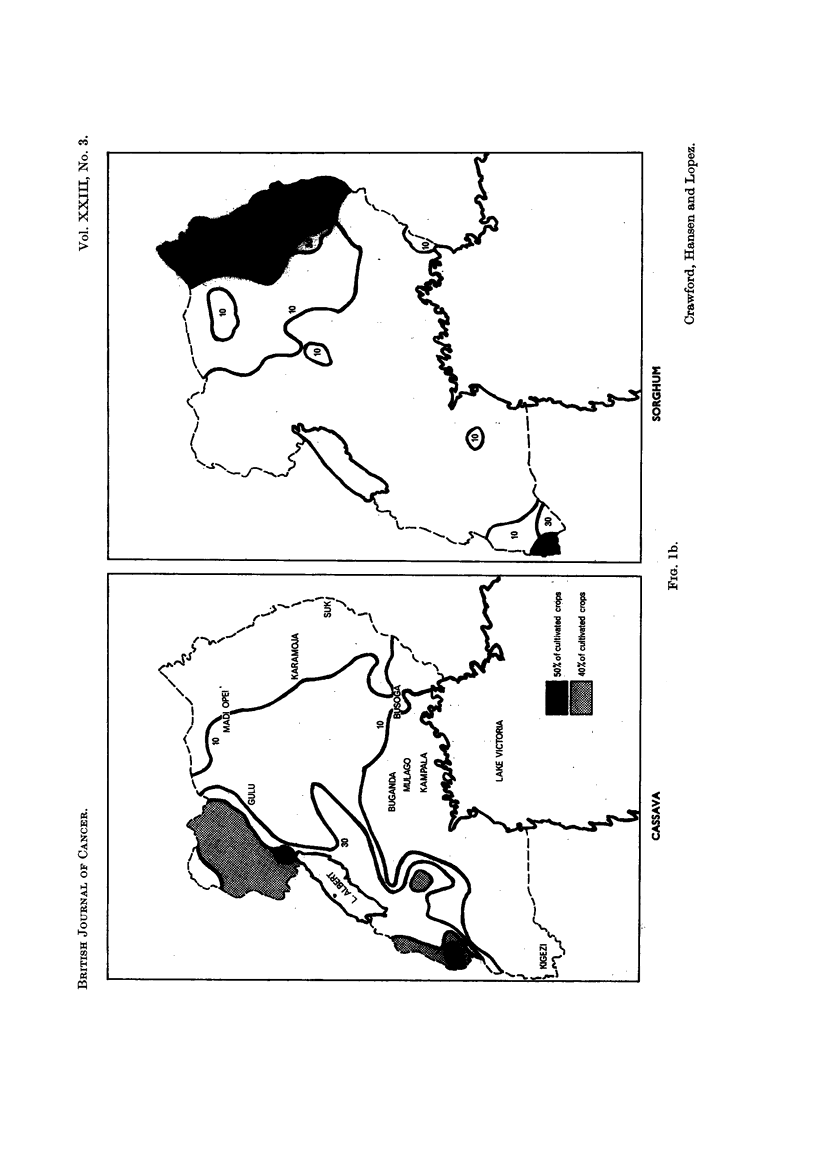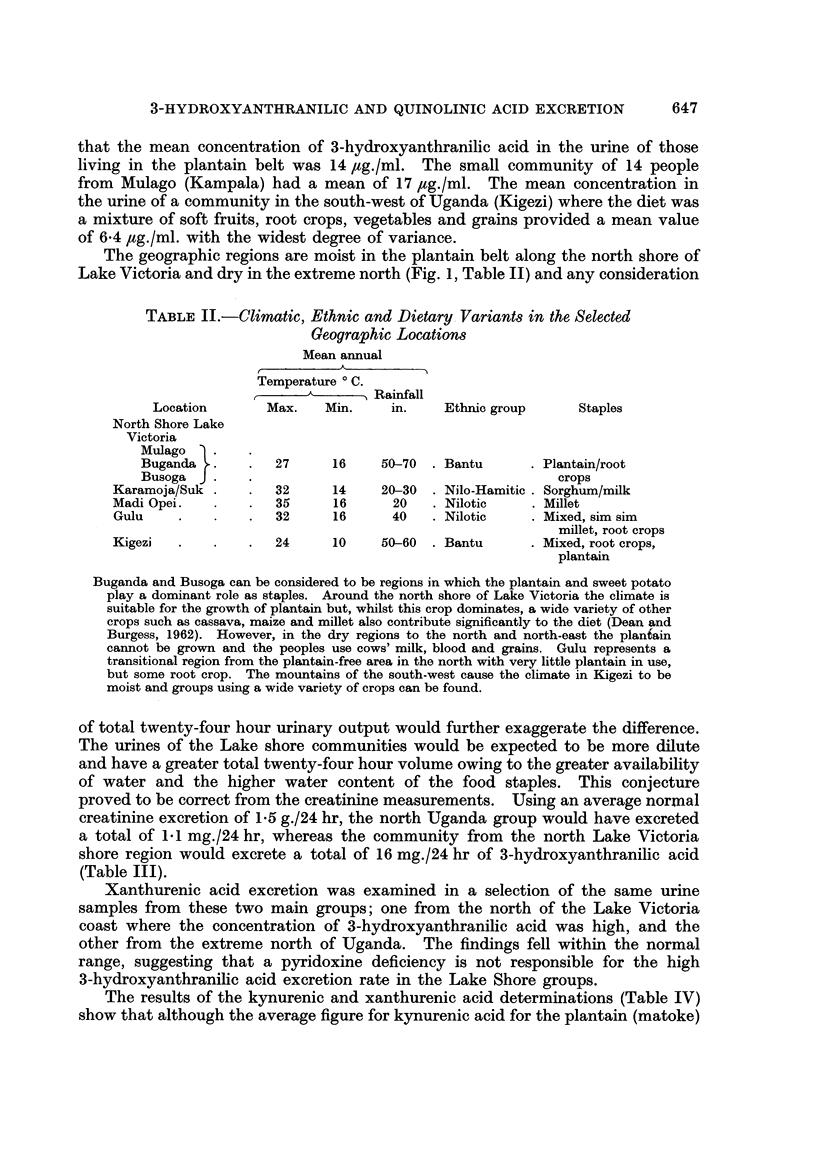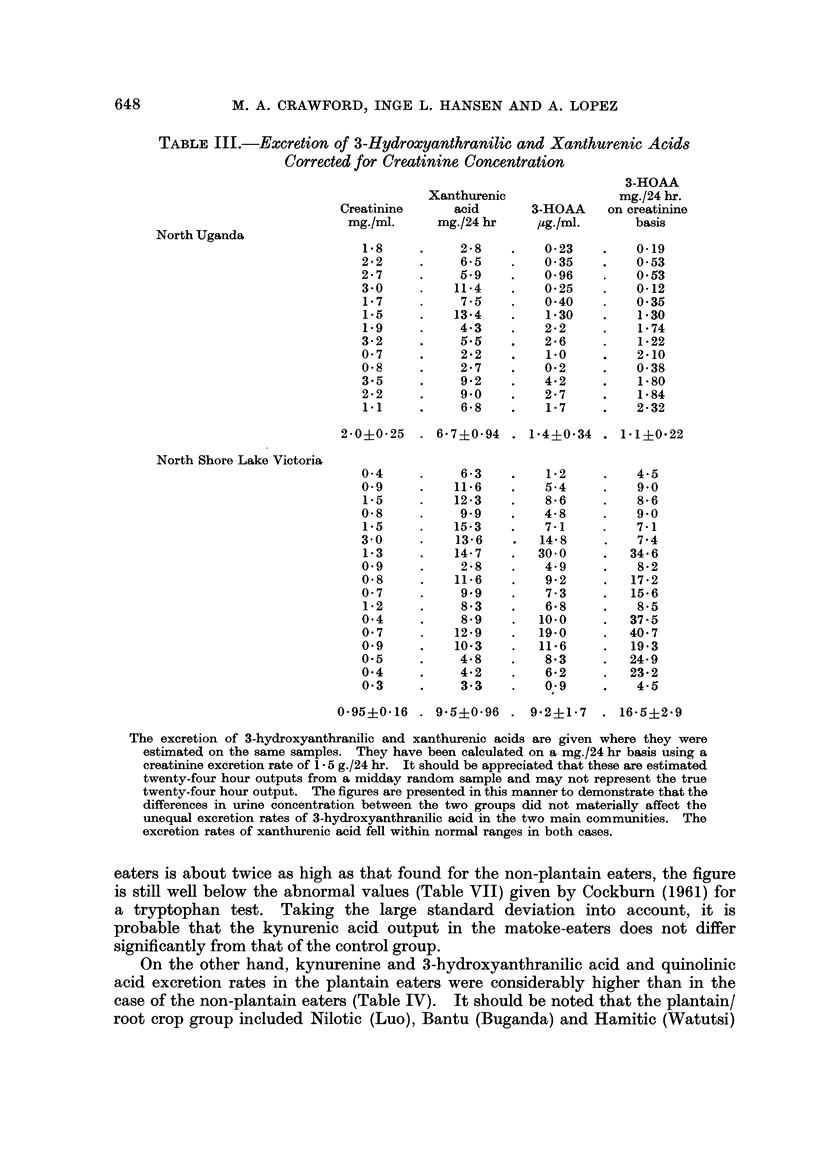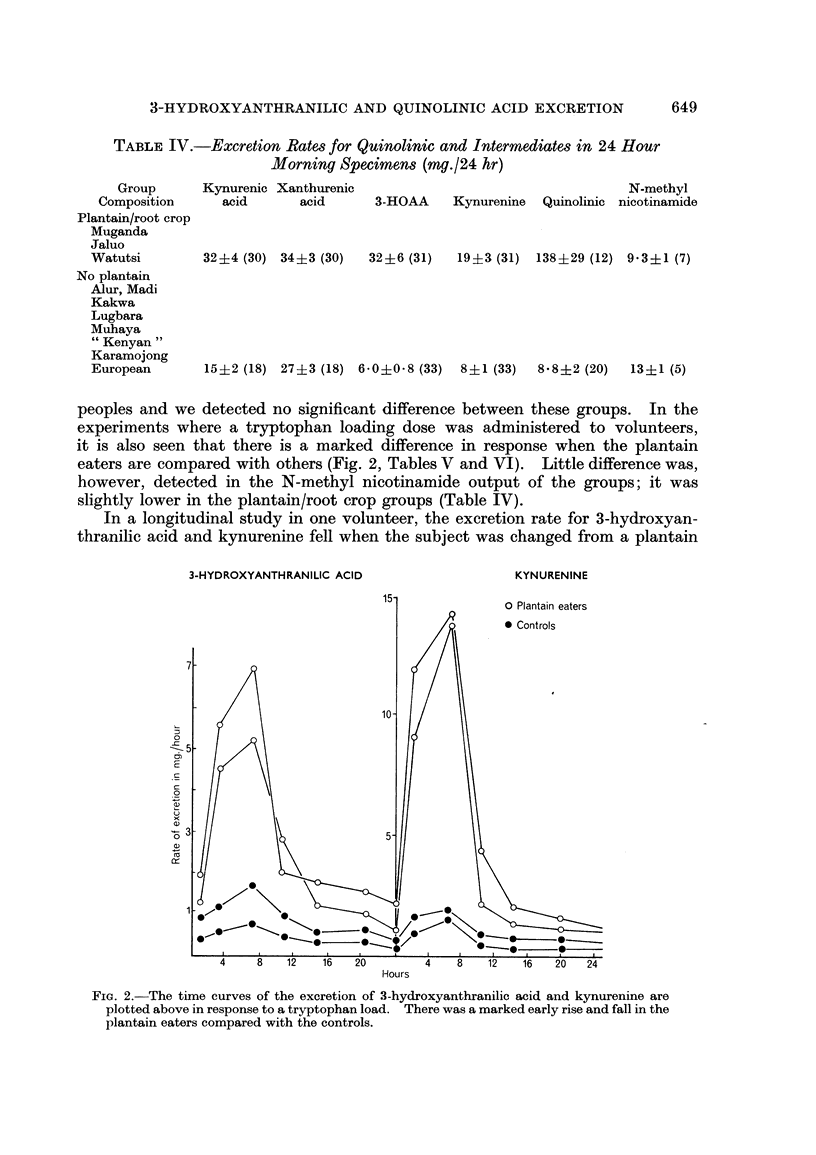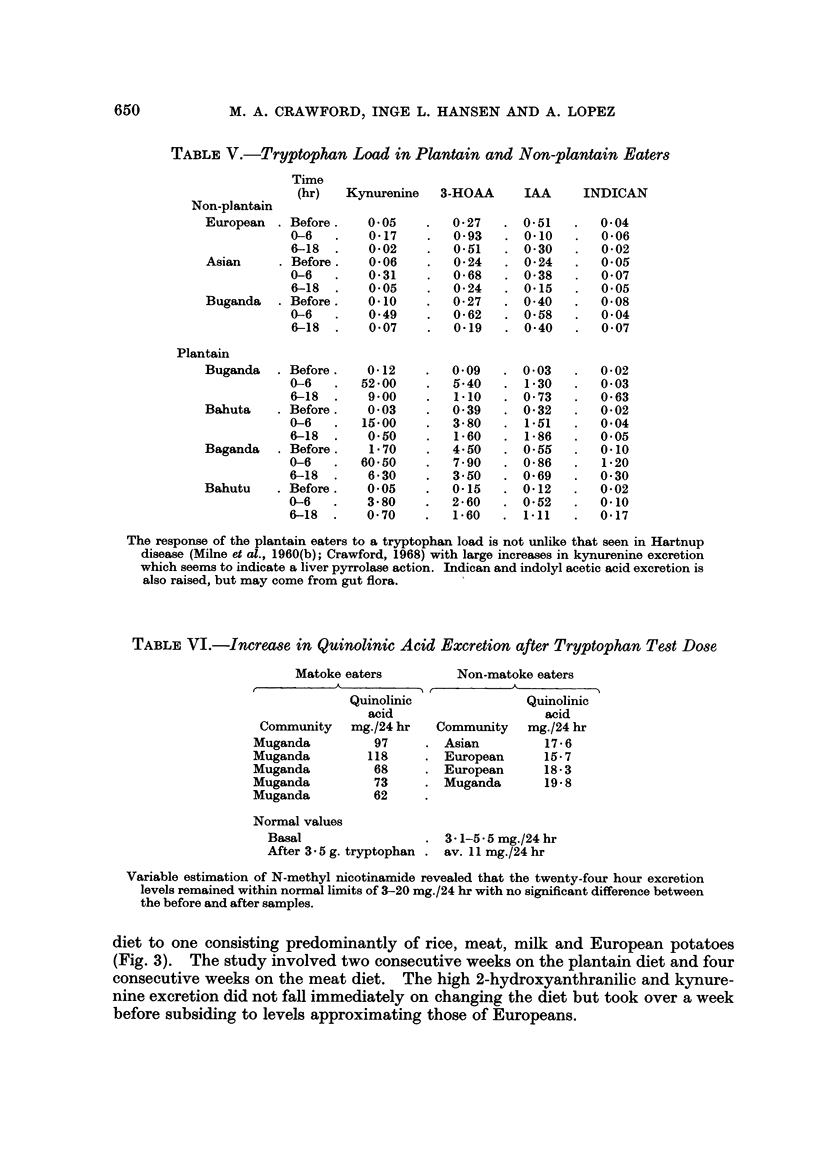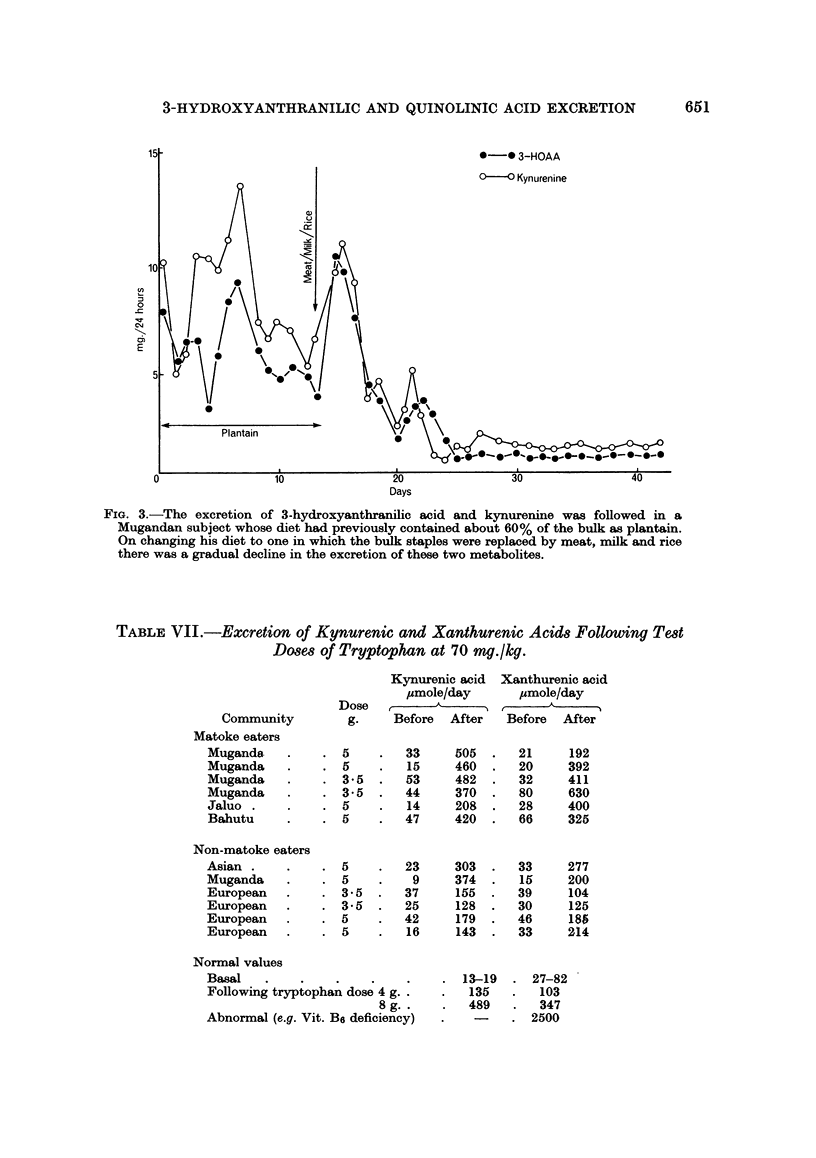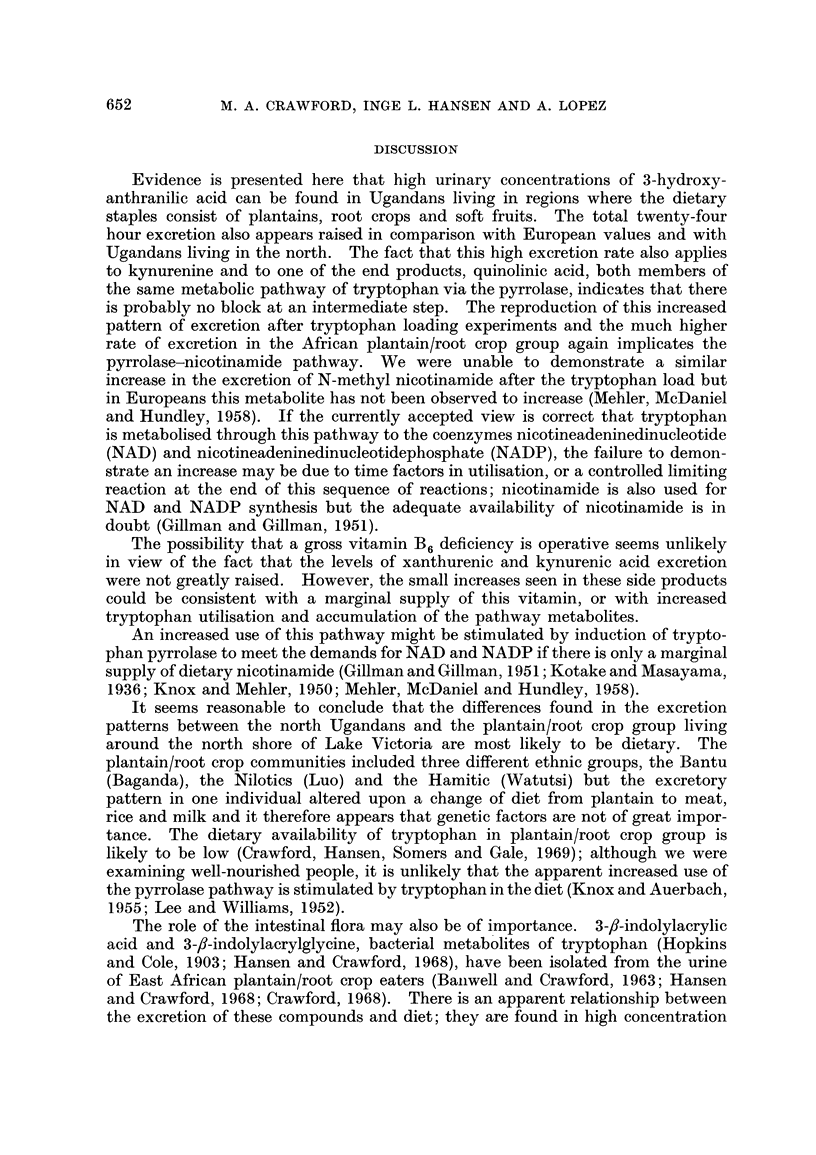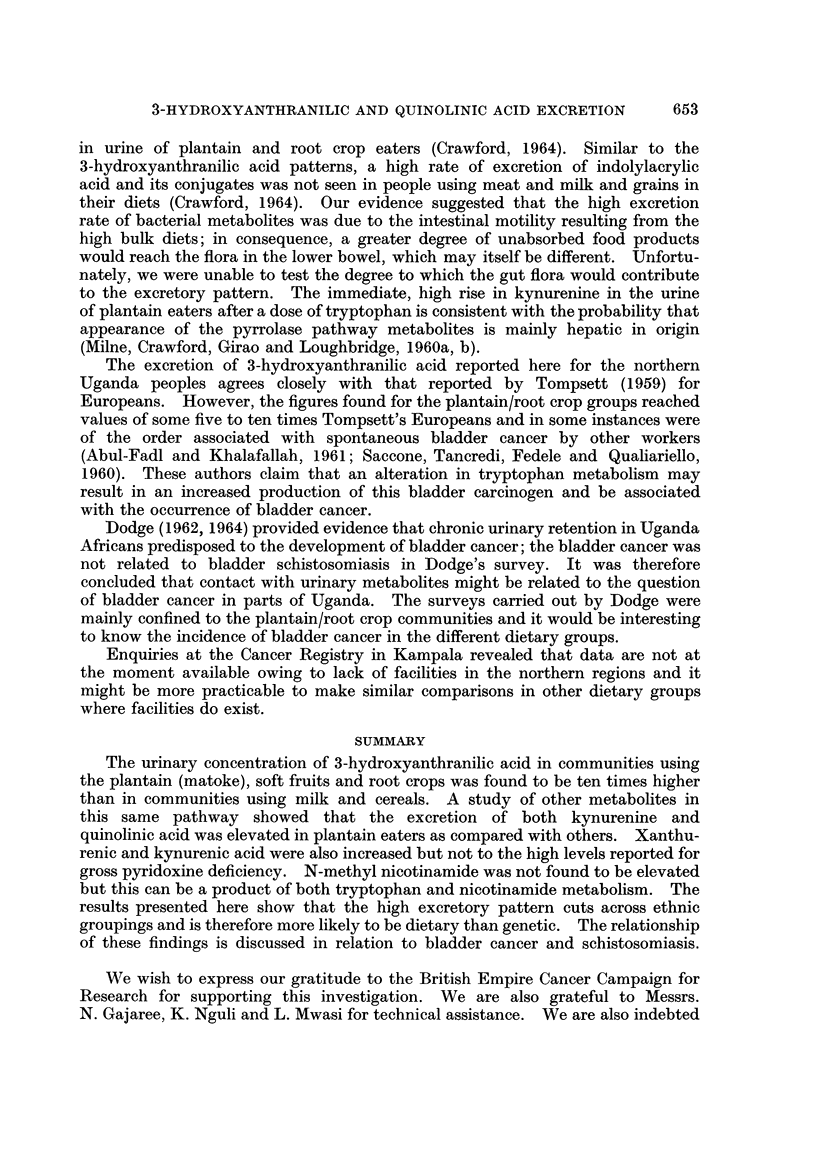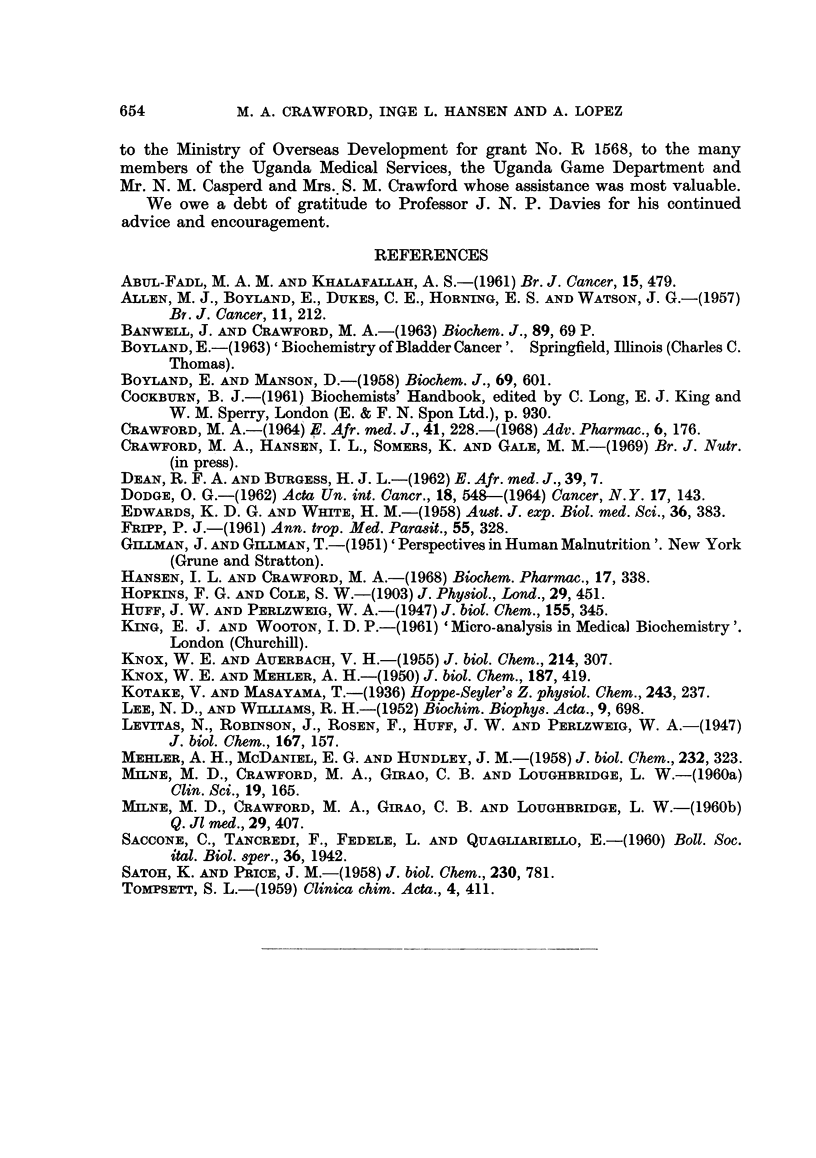# The excretion of 3-hydroxyanthranilic and quinolinic acid in Uganda Africans.

**DOI:** 10.1038/bjc.1969.80

**Published:** 1969-09

**Authors:** M. A. Crawford, I. L. Hansen, A. Lopez


### THE EXCRETION OF 3-HYDROXYANTHRANILIC AND QUINOLINIC ACID IN UGANDA AFRICANS

M. A. CRAWFORD*, INGE L. HANSEN AND A. LOPEZ

*From the Department of Biochemistry, Makerere University, College Medical School, Uganda*

Received for publication June 26, 1969.

EXPERIENCE in the aniline dye industry led to research showing that *ortho*-aminophenols were carcinogenic in experimental animals. Protective measures against contamination of humans in that industry severely reduced the incidence of bladder cancer and it was therefore concluded that the *ortho*-aminophenols are causative of human bladder cancer. The fact that 3-hydroxyanthranilic acid (3-HOAA) is a normal metabolite present in urine and is also an *ortho*-aminophenol led to the demonstration of its carcinogenic properties and the proposition that it may play a role in the development of spontaneous human bladder cancer (Allen, Boyland, Dukes, Horning and Watson, 1957; Boyland and Manson, 1958; Boyland, 1963).

Dodge (1962, 1964) reported on the incidence of bladder cancer in Uganda. Although bladder cancer in the tropics is frequently associated with schistosomiasis, usually *Schistosoma haematobium*, Dodge could not find any evidence for a direct association between schistosomiasis and bladder cancer in the Uganda Africans covered by his survey: bladder schistosomiasis was uncommon. The present study was carried out to assess the urinary concentration of 3-hydroxyanthranilic acid in communities living in different regions of Uganda.

### METHODS

### *Geographical studies*

The strong geographical and climatic contrasts in East Africa make it possible to examine rural communities who by virtue of local custom, climatic and geographical considerations, are restricted to certain types of crops or food staples (see Fig. 1a, b). Two extremes were chosen for this survey; people who live around the northern shore of Lake Victoria (Mulago, Buganda and Busoga) where the plantain (*Musa* sp.) was used as a dominant staple (matoke, i.e. plantain cooked by steaming), and people living in semi-arid areas (Madi Opei) to the extreme north of Uganda. These latter people predominantly use grains such as finger millet (*Eleusine coracana*) and sorghum (*Sorghum vulgare*); they also drink milk from cattle and goats. Collections were also made from a third group in the mountainous region to the south-west (Kigezi) which used mixed staples consisting of root crops, including sweet potatoes (*Ipomea batatas*), plantain and sorghum. Two other subsidiary groups were included in this survey—a semi-arid living community in the north-east (Karamoja/Suk) and a group outside the northern limits of the wet plantain belt on the north lake shore but not sufficiently far

* Present address: M. A. Crawford, Nuffield Institute of Comparative Medicine, The Zoological Society of London, Regent's Park, London, N.W.1.

removed to be considered semi-arid (Gulu). This latter group was intermediate with grain-based communities on one hand and root crop communities on the other. The plantain plays only a small role in the diet of some people in this area. To the north-east it is virtually non-existent; to the south, abundant (Table II and Fig. 1). Consequently, two main groups are discussed in this study, (i) plantain/root crop, and (ii) grains/milk.

Urine samples were collected from clinically healthy young adults in rural communities in different areas of Uganda. On collection, the urines were centrifuged and the sediment examined for parasites; creatinine/urea; reducing substances, protein and specific gravity; the few which were abnormal were discarded.

Small samples were packed with ice and frozen within 4–8 hours of collection and stored at $-15°$ C. until analysed. Twenty-four hour urine samples were collected under toluene, or preserved with dilute hydrochloric acid (King and Wooton, 1961); a 50 ml. aliquot was stored frozen for analysis. Recovery experiments showed that the method of preservation did not affect kynurenic acid, xanthurenic acid and N-methyl nicotinamide determination. Samples collected in dilute acid were analysed immediately for quinolinic acid in order to avoid conversion of the acid labile quinolinic acid to nicotinic acid. Tryptophan loads were administered orally in doses of 20 mg./kg. body weight in the morning. Urine was collected for twenty-four hours after the dose; urine was also collected at two hourly intervals after the dose when an oral water load of approximately 500 ml./2 hr was used to facilitate accurate collection.

*Chemical methods*

The method of Tompsett (1959) was used for 3-hydroxyanthranilic acid and kynurenine. The estimate was made immediately on thawing the urine so that no time was allowed for enzymic breakdown of any conjugates (Fripp, 1961). Kynurenic acid and xanthurenic acid were estimated by fluorimetry after separation on an ion-exchange column. The method used was that described by Satoh and Price (1958) except that the original Dowex-50-column was replaced by a column of amberlite IR-120(H). The recoveries obtained on this column were 88% for kynurenic acid and 77% for xanthurenic acid. Kynurenic acid was activated at 240 m$\mu$ and fluorescence measured at 435 m$\mu$ using a Zeiss spectrofluorimeter. Xanthurenic acid was activated at 370 m$\mu$ and the fluorescence read at 530 m$\mu$.

Quinolinic acid was estimated by measurement of the UV-absorption at the 265 m$\mu$ absorption-maximum after separation from urine by "thick-layer" chromatography. 3 ml. of neat urine are applied to a 2 mm. thick Kieselgel plate (Kieselgel G "nach Stahl"; 20 $\times$ 20 cm.). The plate was run in a solvent-system consisting of ethanol (95%)–ammonia (sp. gr. 0·91) in the proportions of 8 : 2 until the solvent front had migrated about 12 cm. The quinolinic acid concentrated in a zone about 2 cm. from the origin (Rf quinolinic acid = 0·19); a 1 cm. zone around this Rf-value was scraped off into a Buchner funnel (washed filter paper Whatman No. 1) and eluted with 50 ml. of 1% $NH_4OH$. To ensure all quinolinic acid was recovered from the plate, the two adjacent 1 cm. zones were scraped off and eluted separately. The extracts were evaporated under vacuum in a water bath at 100° C. and the residue was taken up in 4 ml. of 1% $NH_4OH$. The UV absorption curve between 295 m$\mu$ was measured with a Zeiss spectrophotometer against a blank obtained by treating a 1 cm. zone from a "blank"

plate. The peak at 265 m$\mu$ was used for quantitative determinations. It was important to ensure that the layer-thickness of " thick-plates " was uniform as the Kieselgel-blank showed a certain amount of ammonia-soluble absorption with maximum at 250–255 m$\mu$ when measured against 1% $NH_4OH$.

The recovery from urine of quinolinic acid standards when treated according to this procedure was 79% $\pm$ 2% (6 experiments), and 78% $\pm$ 5% (6 experiments). None of the samples examined by us (obtained from subjects free of drugs) contained material which directly interfered with the quinolinic acid spectrum.

The method was found valuable for estimation of the high outputs of quinolinic acid in plantain (matoke) eaters. It can also be used for normal outputs provided the urine is not very dilute (lower concentration limit approximately 3 $\mu$g./ml.), but for work with very dilute urines, a microbiological method would probably be preferable. For our purpose, however, the method was satisfactory as the urines were concentrated and the levels of quinolinic acid high.

N-methyl nicotinamide was estimated by fluorimetry according to the method of Huff and Perlzweig (1947), and Levitas, Robinson, Rosen, Huff and Perlzweig (1947), using activation wavelength 365 m$\mu$ and fluorescence wavelength 440 m$\mu$.

Creatinine was measured using the technique described by Edwards and White (1958) in order to provide an assessment of the approximate twenty-four hour output. Xanthurenic acid was measured using the method of Satoh and Price (1958).

### RESULTS

The assays of 3-hydroxyanthranilic acid are expressed as concentrations of free 3-HOAA rather than total excretion rates as it is the concentration of free acid in contact with the bladder wall which would be of importance. Table I shows

TABLE I.—*The Geographic Variation in Excretion of* 3-*Hydroxyanthranilic Acid in Uganda Africans*

| Location | No. of observations | Mean $\mu$g./ml. | Coefficient of variance | Standard error |
|---|---|---|---|---|
| Mulago | 14 | 17·0 | 27 | ±1·2 |
| Buganda/Busoga | 26 | 14·0 | 33 | ±0·9 |
| Karamoja/Suk | 41 | 3·6 | 55 | ±0·3 |
| Madi Opei | 26 | 2·3 | 59 | ±0·3 |
| Gulu | 19 | 5·5 | 58 | ±0·7 |
| Kigezi | 17 | 6·3 | 109 | ±1·6 |

The climatic, ethnic and dietary variants are provided in Table II. The Mulago/Buganda/Busoga communities lie along the north shore of Lake Victoria and the mean concentration of the 3-hydroxyanthranilic acid in the urine is significantly greater than the peoples from the semi-arid country (Karamoja/Suk/Madi Opei) where millet, milk and sorghum constitute dominant staples.

The co-efficient of variance is highest in the Kigezi community; this could be consistent with the fact that contributors were drawn from groups with widely mixed feeding customs.

### EXPLANATION OF PLATES

FIG. 1a and b.—These show the distribution of the most important food crops in Uganda taken from the Uganda Atlas. Isopleth values are drawn in at 10% and 30% cultivation relative to other crops. 40% and 50% and over areas are shaded as in the key. As the Uganda Atlas admits, these divisions can only be approximations but they nonetheless clearly illustrate the different use of principal food materials in different regions of Uganda.

FIG. 1a.

Crawford, Hansen and Lupez.

Fig. 1b.

Crawford, Hansen and Lopez.

that the mean concentration of 3-hydroxyanthranilic acid in the urine of those living in the plantain belt was 14 $\mu$g./ml. The small community of 14 people from Mulago (Kampala) had a mean of 17 $\mu$g./ml. The mean concentration in the urine of a community in the south-west of Uganda (Kigezi) where the diet was a mixture of soft fruits, root crops, vegetables and grains provided a mean value of 6·4 $\mu$g./ml. with the widest degree of variance.

The geographic regions are moist in the plantain belt along the north shore of Lake Victoria and dry in the extreme north (Fig. 1, Table II) and any consideration

TABLE II.—*Climatic, Ethnic and Dietary Variants in the Selected Geographic Locations*

| Location | Mean annual Temperature ° C. Max. | Min. | Rainfall in. | Ethnic group | Staples |
|---|---|---|---|---|---|
| North Shore Lake Victoria Mulago, Buganda, Busoga | 27 | 16 | 50–70 | Bantu | Plantain/root crops |
| Karamoja/Suk | 32 | 14 | 20–30 | Nilo-Hamitic | Sorghum/milk |
| Madi Opei | 35 | 16 | 20 | Nilotic | Millet |
| Gulu | 32 | 16 | 40 | Nilotic | Mixed, sim sim millet, root crops |
| Kigezi | 24 | 10 | 50–60 | Bantu | Mixed, root crops, plantain |

Buganda and Busoga can be considered to be regions in which the plantain and sweet potato play a dominant role as staples. Around the north shore of Lake Victoria the climate is suitable for the growth of plantain but, whilst this crop dominates, a wide variety of other crops such as cassava, maize and millet also contribute significantly to the diet (Dean and Burgess, 1962). However, in the dry regions to the north and north-east the plantain cannot be grown and the peoples use cows' milk, blood and grains. Gulu represents a transitional region from the plantain-free area in the north with very little plantain in use, but some root crop. The mountains of the south-west cause the climate in Kigezi to be moist and groups using a wide variety of crops can be found.

of total twenty-four hour urinary output would further exaggerate the difference. The urines of the Lake shore communities would be expected to be more dilute and have a greater total twenty-four hour volume owing to the greater availability of water and the higher water content of the food staples. This conjecture proved to be correct from the creatinine measurements. Using an average normal creatinine excretion of 1·5 g./24 hr, the north Uganda group would have excreted a total of 1·1 mg./24 hr, whereas the community from the north Lake Victoria shore region would excrete a total of 16 mg./24 hr of 3-hydroxyanthranilic acid (Table III).

Xanthurenic acid excretion was examined in a selection of the same urine samples from these two main groups; one from the north of the Lake Victoria coast where the concentration of 3-hydroxyanthranilic acid was high, and the other from the extreme north of Uganda. The findings fell within the normal range, suggesting that a pyridoxine deficiency is not responsible for the high 3-hydroxyanthranilic acid excretion rate in the Lake Shore groups.

The results of the kynurenic and xanthurenic acid determinations (Table IV) show that although the average figure for kynurenic acid for the plantain (matoke)

TABLE III.—*Excretion of 3-Hydroxyanthranilic and Xanthurenic Acids Corrected for Creatinine Concentration*

| | Creatinine mg./ml. | Xanthurenic acid mg./24 hr | 3-HOAA µg./ml. | 3-HOAA mg./24 hr. on creatinine basis |
|---|---|---|---|---|
| North Uganda | | | | |
| | 1·8 | 2·8 | 0·23 | 0·19 |
| | 2·2 | 6·5 | 0·35 | 0·53 |
| | 2·7 | 5·9 | 0·96 | 0·53 |
| | 3·0 | 11·4 | 0·25 | 0·12 |
| | 1·7 | 7·5 | 0·40 | 0·35 |
| | 1·5 | 13·4 | 1·30 | 1·30 |
| | 1·9 | 4·3 | 2·2 | 1·74 |
| | 3·2 | 5·5 | 2·6 | 1·22 |
| | 0·7 | 2·2 | 1·0 | 2·10 |
| | 0·8 | 2·7 | 0·2 | 0·38 |
| | 3·5 | 9·2 | 4·2 | 1·80 |
| | 2·2 | 9·0 | 2·7 | 1·84 |
| | 1·1 | 6·8 | 1·7 | 2·32 |
| | 2·0±0·25 | 6·7±0·94 | 1·4±0·34 | 1·1±0·22 |
| North Shore Lake Victoria | | | | |
| | 0·4 | 6·3 | 1·2 | 4·5 |
| | 0·9 | 11·6 | 5·4 | 9·0 |
| | 1·5 | 12·3 | 8·6 | 8·6 |
| | 0·8 | 9·9 | 4·8 | 9·0 |
| | 1·5 | 15·3 | 7·1 | 7·1 |
| | 3·0 | 13·6 | 14·8 | 7·4 |
| | 1·3 | 14·7 | 30·0 | 34·6 |
| | 0·9 | 2·8 | 4·9 | 8·2 |
| | 0·8 | 11·6 | 9·2 | 17·2 |
| | 0·7 | 9·9 | 7·3 | 15·6 |
| | 1·2 | 8·3 | 6·8 | 8·5 |
| | 0·4 | 8·9 | 10·0 | 37·5 |
| | 0·7 | 12·9 | 19·0 | 40·7 |
| | 0·9 | 10·3 | 11·6 | 19·3 |
| | 0·5 | 4·8 | 8·3 | 24·9 |
| | 0·4 | 4·2 | 6·2 | 23·2 |
| | 0·3 | 3·3 | 0·9 | 4·5 |
| | 0·95±0·16 | 9·5±0·96 | 9·2±1·7 | 16·5±2·9 |

The excretion of 3-hydroxyanthranilic and xanthurenic acids are given where they were estimated on the same samples. They have been calculated on a mg./24 hr basis using a creatinine excretion rate of 1·5 g./24 hr. It should be appreciated that these are estimated twenty-four hour outputs from a midday random sample and may not represent the true twenty-four hour output. The figures are presented in this manner to demonstrate that the differences in urine concentration between the two groups did not materially affect the unequal excretion rates of 3-hydroxyanthranilic acid in the two main communities. The excretion rates of xanthurenic acid fell within normal ranges in both cases.

eaters is about twice as high as that found for the non-plantain eaters, the figure is still well below the abnormal values (Table VII) given by Cockburn (1961) for a tryptophan test. Taking the large standard deviation into account, it is probable that the kynurenic acid output in the matoke-eaters does not differ significantly from that of the control group.

On the other hand, kynurenine and 3-hydroxyanthranilic acid and quinolinic acid excretion rates in the plantain eaters were considerably higher than in the case of the non-plantain eaters (Table IV). It should be noted that the plantain/root crop group included Nilotic (Luo), Bantu (Buganda) and Hamitic (Watutsi)

TABLE IV.—*Excretion Rates for Quinolinic and Intermediates in 24 Hour Morning Specimens (mg./24 hr)*

| Group Composition | Kynurenic acid | Xanthurenic acid | 3-HOAA | Kynurenine | Quinolinic | N-methyl nicotinamide |
|---|---|---|---|---|---|---|
| Plantain/root crop<br>Muganda<br>Jaluo<br>Watutsi | 32±4 (30) | 34±3 (30) | 32±6 (31) | 19±3 (31) | 138±29 (12) | 9·3±1 (7) |
| No plantain<br>Alur, Madi<br>Kakwa<br>Lugbara<br>Muhaya<br>" Kenyan "<br>Karamojong<br>European | 15±2 (18) | 27±3 (18) | 6·0±0·8 (33) | 8±1 (33) | 8·8±2 (20) | 13±1 (5) |

peoples and we detected no significant difference between these groups. In the experiments where a tryptophan loading dose was administered to volunteers, it is also seen that there is a marked difference in response when the plantain eaters are compared with others (Fig. 2, Tables V and VI). Little difference was, however, detected in the N-methyl nicotinamide output of the groups; it was slightly lower in the plantain/root crop groups (Table IV).

In a longitudinal study in one volunteer, the excretion rate for 3-hydroxyanthranilic acid and kynurenine fell when the subject was changed from a plantain

FIG. 2.—The time curves of the excretion of 3-hydroxyanthranilic acid and kynurenine are plotted above in response to a tryptophan load. There was a marked early rise and fall in the plantain eaters compared with the controls.

TABLE V.—*Tryptophan Load in Plantain and Non-plantain Eaters*

| | Time (hr) | Kynurenine | 3-HOAA | IAA | INDICAN |
|---|---|---|---|---|---|
| Non-plantain | | | | | |
| European | Before | 0·05 | 0·27 | 0·51 | 0·04 |
| | 0–6 | 0·17 | 0·93 | 0·10 | 0·06 |
| | 6–18 | 0·02 | 0·51 | 0·30 | 0·02 |
| Asian | Before | 0·06 | 0·24 | 0·24 | 0·05 |
| | 0–6 | 0·31 | 0·68 | 0·38 | 0·07 |
| | 6–18 | 0·05 | 0·24 | 0·15 | 0·05 |
| Buganda | Before | 0·10 | 0·27 | 0·40 | 0·08 |
| | 0–6 | 0·49 | 0·62 | 0·58 | 0·04 |
| | 6–18 | 0·07 | 0·19 | 0·40 | 0·07 |
| Plantain | | | | | |
| Buganda | Before | 0·12 | 0·09 | 0·03 | 0·02 |
| | 0–6 | 52·00 | 5·40 | 1·30 | 0·03 |
| | 6–18 | 9·00 | 1·10 | 0·73 | 0·63 |
| Bahuta | Before | 0·03 | 0·39 | 0·32 | 0·02 |
| | 0–6 | 15·00 | 3·80 | 1·51 | 0·04 |
| | 6–18 | 0·50 | 1·60 | 1·86 | 0·05 |
| Baganda | Before | 1·70 | 4·50 | 0·55 | 0·10 |
| | 0–6 | 60·50 | 7·90 | 0·86 | 1·20 |
| | 6–18 | 6·30 | 3·50 | 0·69 | 0·30 |
| Bahutu | Before | 0·05 | 0·15 | 0·12 | 0·02 |
| | 0–6 | 3·80 | 2·60 | 0·52 | 0·10 |
| | 6–18 | 0·70 | 1·60 | 1·11 | 0·17 |

The response of the plantain eaters to a tryptophan load is not unlike that seen in Hartnup disease (Milne *et al.*, 1960(b); Crawford, 1968) with large increases in kynurenine excretion which seems to indicate a liver pyrrolase action. Indican and indolyl acetic acid excretion is also raised, but may come from gut flora.

TABLE VI.—*Increase in Quinolinic Acid Excretion after Tryptophan Test Dose*

| Matoke eaters | | Non-matoke eaters | |
|---|---|---|---|
| Community | Quinolinic acid mg./24 hr | Community | Quinolinic acid mg./24 hr |
| Muganda | 97 | Asian | 17·6 |
| Muganda | 118 | European | 15·7 |
| Muganda | 68 | European | 18·3 |
| Muganda | 73 | Muganda | 19·8 |
| Muganda | 62 | | |

Normal values

| | |
|---|---|
| Basal | 3·1–5·5 mg./24 hr |
| After 3·5 g. tryptophan | av. 11 mg./24 hr |

Variable estimation of N-methyl nicotinamide revealed that the twenty-four hour excretion levels remained within normal limits of 3–20 mg./24 hr with no significant difference between the before and after samples.

diet to one consisting predominantly of rice, meat, milk and European potatoes (Fig. 3). The study involved two consecutive weeks on the plantain diet and four consecutive weeks on the meat diet. The high 2-hydroxyanthranilic and kynurenine excretion did not fall immediately on changing the diet but took over a week before subsiding to levels approximating those of Europeans.

FIG. 3.—The excretion of 3-hydroxyanthranilic acid and kynurenine was followed in a Mugandan subject whose diet had previously contained about 60% of the bulk as plantain. On changing his diet to one in which the bulk staples were replaced by meat, milk and rice there was a gradual decline in the excretion of these two metabolites.

TABLE VII.—*Excretion of Kynurenic and Xanthurenic Acids Following Test Doses of Tryptophan at 70 mg./kg.*

| Community | Dose g. | Kynurenic acid µmole/day Before | Kynurenic acid µmole/day After | Xanthurenic acid µmole/day Before | Xanthurenic acid µmole/day After |
|---|---|---|---|---|---|
| Matoke eaters | | | | | |
| Muganda | 5 | 33 | 505 | 21 | 192 |
| Muganda | 5 | 15 | 460 | 20 | 392 |
| Muganda | 3·5 | 53 | 482 | 32 | 411 |
| Muganda | 3·5 | 44 | 370 | 80 | 630 |
| Jaluo | 5 | 14 | 208 | 28 | 400 |
| Bahutu | 5 | 47 | 420 | 66 | 325 |
| Non-matoke eaters | | | | | |
| Asian | 5 | 23 | 303 | 33 | 277 |
| Muganda | 5 | 9 | 374 | 15 | 200 |
| European | 3·5 | 37 | 155 | 39 | 104 |
| European | 3·5 | 25 | 128 | 30 | 125 |
| European | 5 | 42 | 179 | 46 | 185 |
| European | 5 | 16 | 143 | 33 | 214 |
| Normal values | | | | | |
| Basal | | | 13–19 | 27–82 | |
| Following tryptophan dose 4 g. | | | 135 | 103 | |
| 8 g. | | | 489 | 347 | |
| Abnormal (*e.g.* Vit. $B_6$ deficiency) | | | — | 2500 | |

### DISCUSSION

Evidence is presented here that high urinary concentrations of 3-hydroxy-anthranilic acid can be found in Ugandans living in regions where the dietary staples consist of plantains, root crops and soft fruits. The total twenty-four hour excretion also appears raised in comparison with European values and with Ugandans living in the north. The fact that this high excretion rate also applies to kynurenine and to one of the end products, quinolinic acid, both members of the same metabolic pathway of tryptophan via the pyrrolase, indicates that there is probably no block at an intermediate step. The reproduction of this increased pattern of excretion after tryptophan loading experiments and the much higher rate of excretion in the African plantain/root crop group again implicates the pyrrolase–nicotinamide pathway. We were unable to demonstrate a similar increase in the excretion of N-methyl nicotinamide after the tryptophan load but in Europeans this metabolite has not been observed to increase (Mehler, McDaniel and Hundley, 1958). If the currently accepted view is correct that tryptophan is metabolised through this pathway to the coenzymes nicotineadeninedinucleotide (NAD) and nicotineadeninedinucleotidephosphate (NADP), the failure to demonstrate an increase may be due to time factors in utilisation, or a controlled limiting reaction at the end of this sequence of reactions; nicotinamide is also used for NAD and NADP synthesis but the adequate availability of nicotinamide is in doubt (Gillman and Gillman, 1951).

The possibility that a gross vitamin $B_6$ deficiency is operative seems unlikely in view of the fact that the levels of xanthurenic and kynurenic acid excretion were not greatly raised. However, the small increases seen in these side products could be consistent with a marginal supply of this vitamin, or with increased tryptophan utilisation and accumulation of the pathway metabolites.

An increased use of this pathway might be stimulated by induction of tryptophan pyrrolase to meet the demands for NAD and NADP if there is only a marginal supply of dietary nicotinamide (Gillman and Gillman, 1951; Kotake and Masayama, 1936; Knox and Mehler, 1950; Mehler, McDaniel and Hundley, 1958).

It seems reasonable to conclude that the differences found in the excretion patterns between the north Ugandans and the plantain/root crop group living around the north shore of Lake Victoria are most likely to be dietary. The plantain/root crop communities included three different ethnic groups, the Bantu (Baganda), the Nilotics (Luo) and the Hamitic (Watutsi) but the excretory pattern in one individual altered upon a change of diet from plantain to meat, rice and milk and it therefore appears that genetic factors are not of great importance. The dietary availability of tryptophan in plantain/root crop group is likely to be low (Crawford, Hansen, Somers and Gale, 1969); although we were examining well-nourished people, it is unlikely that the apparent increased use of the pyrrolase pathway is stimulated by tryptophan in the diet (Knox and Auerbach, 1955; Lee and Williams, 1952).

The role of the intestinal flora may also be of importance. 3-$\beta$-indolylacrylic acid and 3-$\beta$-indolylacrylglycine, bacterial metabolites of tryptophan (Hopkins and Cole, 1903; Hansen and Crawford, 1968), have been isolated from the urine of East African plantain/root crop eaters (Banwell and Crawford, 1963; Hansen and Crawford, 1968; Crawford, 1968). There is an apparent relationship between the excretion of these compounds and diet; they are found in high concentration in urine of plantain and root crop eaters (Crawford, 1964). Similar to the 3-hydroxyanthranilic acid patterns, a high rate of excretion of indolylacrylic acid and its conjugates was not seen in people using meat and milk and grains in their diets (Crawford, 1964). Our evidence suggested that the high excretion rate of bacterial metabolites was due to the intestinal motility resulting from the high bulk diets; in consequence, a greater degree of unabsorbed food products would reach the flora in the lower bowel, which may itself be different. Unfortunately, we were unable to test the degree to which the gut flora would contribute to the excretory pattern. The immediate, high rise in kynurenine in the urine of plantain eaters after a dose of tryptophan is consistent with the probability that appearance of the pyrrolase pathway metabolites is mainly hepatic in origin (Milne, Crawford, Girao and Loughbridge, 1960a, b).

The excretion of 3-hydroxyanthranilic acid reported here for the northern Uganda peoples agrees closely with that reported by Tompsett (1959) for Europeans. However, the figures found for the plantain/root crop groups reached values of some five to ten times Tompsett's Europeans and in some instances were of the order associated with spontaneous bladder cancer by other workers (Abul-Fadl and Khalafallah, 1961; Saccone, Tancredi, Fedele and Qualiariello, 1960). These authors claim that an alteration in tryptophan metabolism may result in an increased production of this bladder carcinogen and be associated with the occurrence of bladder cancer.

Dodge (1962, 1964) provided evidence that chronic urinary retention in Uganda Africans predisposed to the development of bladder cancer; the bladder cancer was not related to bladder schistosomiasis in Dodge's survey. It was therefore concluded that contact with urinary metabolites might be related to the question of bladder cancer in parts of Uganda. The surveys carried out by Dodge were mainly confined to the plantain/root crop communities and it would be interesting to know the incidence of bladder cancer in the different dietary groups.

Enquiries at the Cancer Registry in Kampala revealed that data are not at the moment available owing to lack of facilities in the northern regions and it might be more practicable to make similar comparisons in other dietary groups where facilities do exist.

### SUMMARY

The urinary concentration of 3-hydroxyanthranilic acid in communities using the plantain (matoke), soft fruits and root crops was found to be ten times higher than in communities using milk and cereals. A study of other metabolites in this same pathway showed that the excretion of both kynurenine and quinolinic acid was elevated in plantain eaters as compared with others. Xanthurenic and kynurenic acid were also increased but not to the high levels reported for gross pyridoxine deficiency. N-methyl nicotinamide was not found to be elevated but this can be a product of both tryptophan and nicotinamide metabolism. The results presented here show that the high excretory pattern cuts across ethnic groupings and is therefore more likely to be dietary than genetic. The relationship of these findings is discussed in relation to bladder cancer and schistosomiasis.

We wish to express our gratitude to the British Empire Cancer Campaign for Research for supporting this investigation. We are also grateful to Messrs. N. Gajaree, K. Nguli and L. Mwasi for technical assistance. We are also indebted to the Ministry of Overseas Development for grant No. R 1568, to the many members of the Uganda Medical Services, the Uganda Game Department and Mr. N. M. Casperd and Mrs. S. M. Crawford whose assistance was most valuable.

We owe a debt of gratitude to Professor J. N. P. Davies for his continued advice and encouragement.

### REFERENCES

ABUL-FADL, M. A. M. AND KHALAFALLAH, A. S.—(1961) *Br. J. Cancer*, **15**, 479.
ALLEN, M. J., BOYLAND, E., DUKES, C. E., HORNING, E. S. AND WATSON, J. G.—(1957) *Br. J. Cancer*, **11**, 212.
BANWELL, J. AND CRAWFORD, M. A.—(1963) *Biochem. J.*, **89**, 69 P.
BOYLAND, E.—(1963) ' Biochemistry of Bladder Cancer '. Springfield, Illinois (Charles C. Thomas).
BOYLAND, E. AND MANSON, D.—(1958) *Biochem. J.*, **69**, 601.
COCKBURN, B. J.—(1961) Biochemists' Handbook, edited by C. Long, E. J. King and W. M. Sperry, London (E. & F. N. Spon Ltd.), p. 930.
CRAWFORD, M. A.—(1964) *E. Afr. med. J.*, **41**, 228.—(1968) *Adv. Pharmac.*, **6**, 176.
CRAWFORD, M. A., HANSEN, I. L., SOMERS, K. AND GALE, M. M.—(1969) *Br. J. Nutr.* (in press).
DEAN, R. F. A. AND BURGESS, H. J. L.—(1962) *E. Afr. med. J.*, **39**, 7.
DODGE, O. G.—(1962) *Acta Un. int. Cancr.*, **18**, 548—(1964) *Cancer, N.Y.* **17**, 143.
EDWARDS, K. D. G. AND WHITE, H. M.—(1958) *Aust. J. exp. Biol. med. Sci.*, **36**, 383.
FRIPP, P. J.—(1961) *Ann. trop. Med. Parasit.*, **55**, 328.
GILLMAN, J. AND GILLMAN, T.—(1951) ' Perspectives in Human Malnutrition '. New York (Grune and Stratton).
HANSEN, I. L. AND CRAWFORD, M. A.—(1968) *Biochem. Pharmac.*, **17**, 338.
HOPKINS, F. G. AND COLE, S. W.—(1903) *J. Physiol., Lond.*, **29**, 451.
HUFF, J. W. AND PERLZWEIG, W. A.—(1947) *J. biol. Chem.*, **155**, 345.
KING, E. J. AND WOOTON, I. D. P.—(1961) ' Micro-analysis in Medical Biochemistry '. London (Churchill).
KNOX, W. E. AND AUERBACH, V. H.—(1955) *J. biol. Chem.*, **214**, 307.
KNOX, W. E. AND MEHLER, A. H.—(1950) *J. biol. Chem.*, **187**, 419.
KOTAKE, V. AND MASAYAMA, T.—(1936) *Hoppe-Seyler's Z. physiol. Chem.*, **243**, 237.
LEE, N. D., AND WILLIAMS, R. H.—(1952) *Biochim. Biophys. Acta.*, **9**, 698.
LEVITAS, N., ROBINSON, J., ROSEN, F., HUFF, J. W. AND PERLZWEIG, W. A.—(1947) *J. biol. Chem.*, **167**, 157.
MEHLER, A. H., McDANIEL, E. G. AND HUNDLEY, J. M.—(1958) *J. biol. Chem.*, **232**, 323.
MILNE, M. D., CRAWFORD, M. A., GIRAO, C. B. AND LOUGHBRIDGE, L. W.—(1960a) *Clin. Sci.*, **19**, 165.
MILNE, M. D., CRAWFORD, M. A., GIRAO, C. B. AND LOUGHBRIDGE, L. W.—(1960b) *Q. Jl med.*, **29**, 407.
SACCONE, C., TANCREDI, F., FEDELE, L. AND QUAGLIARIELLO, E.—(1960) *Boll. Soc. ital. Biol. sper.*, **36**, 1942.
SATOH, K. AND PRICE, J. M.—(1958) *J. biol. Chem.*, **230**, 781.
TOMPSETT, S. L.—(1959) *Clinica chim. Acta.*, **4**, 411.